# Identification and suppression of low-frequency oscillations using PMU measurements based power system model in smart grid

**DOI:** 10.1038/s41598-025-88389-3

**Published:** 2025-01-30

**Authors:** Mohd Zuhaib, Mohd Rihan, Saket Gupta, Marwan Ahmad Abdullah Sufyan

**Affiliations:** 1 Department of Electrical Engineering, GLA University, Mathura, Uttar Pradesh India; 2 Department of Electrical Engineering, Aligarh Muslim University, Aligarh, Uttar Pradesh India; 3 ICE Department, Bharati Vidyapeeth’s College of Engineering, New Delhi, 110063 India; 4 College of Engineering and Information Technology, Aljanad University of Science and Technology, Taiz, Yemen

**Keywords:** Smart Grid, Low-frequency oscillations, Phasor Measurement Unit (PMU), Power system stabilizer, Wide Area Damping Controller, Etc, Electrical and electronic engineering, Energy infrastructure

## Abstract

Low-frequency oscillations (LFO) are inherent to large interconnected power systems. Timely detection and mitigation of these oscillations is essential to maintain reliable power system operation. This paper presents a methodology to identify and mitigate low-frequency oscillations ( forced and inter-area) using a wide area monitoring system (WAMS) based power system model utilizing phasor measurement units (PMUs). These models accurately identify the behavior and location of generators contributing to low-frequency oscillations in real-time and hence can efficiently improve the performance of WADC to mitigate them. The proportional resonant power system stabilizer (PR-PSS) is utilized to suppress these LFOs, as determined from the Wide Area Power System Model. The damping structure based on PR-PSS with measurements from WAMS effectively suppresses both forced and inter-area oscillation modes.

## Introduction

Low-frequency oscillations (LFOs) are becoming crucial in large interconnected smart grids. They do not threaten power system stability until sufficient positive damping^1^. However, Negatively damped LFOs are of concern and should be detected and damped in real time to avoid power system failure^2^. These oscillations can be categorized as forced oscillations (FO) and inter-area oscillations. Forced oscillations result from generator trips and control actions causing rapid imbalances, malfunction of PSS, mechanical oscillations of generator turbines, Poorly designed PSS, and mistuning/execution of generator controller. In contrast, inter-area oscillations oscillation occurs from random load fluctuations, changes in operating conditions, faults, line tripping, generation loss, etc^2^. The existence of such weakly damped oscillations sometimes results in a cascaded operation that may even lead to a blackout^3^.

These oscillations are frequently observed in Indian, American, and Chinese power grids and are sustained for long, even for hours^4^. Such oscillations may endanger the stability of the power system or damage the essential power system components if an effective suppression technique is not adopted to dampen these oscillations^5^. This has gained the attention of power engineers and researchers to develop an efficient framework to locate the source of these oscillations and mitigate them^6,7^. The idea of WAMS receiving data from PMUs from multiple locations is utilized in several kinds of literature to aid the PSS in suppressing these low-frequency oscillations^8^.

Several works are available in the literature for locating the source of forced oscillations in the power system to ease corrective actions. A prony analysis method using generator-damping torque coefficients to locate oscillation sources is proposed in^9^. Prony and first-wave algorithms explain oscillation phasors in^10^. FO originates from the generator set with the leading phasor. Ref^11^. uses Bayesian numerical methods to identify the source of FO. Ref^12^. utilizes Thomson’s multitaper spectral estimation and harmonic analysis to reduce FO-induced bias in ambient noise spectrum estimation. Ref^13^. suggest locating the FO at the branch level and utilizing deep learning and dissipating energy flow method. In^14^, a grid vulnerability analysis technique is proposed that highlights the areas critical to FOs.

Various advanced signal-processing techniques have also been utilized to identify inter-area oscillations. The basic methods include Fast Fourier Transform (FFT), Matrix Pencil, vector fitting, heuristic algorithms^15,16^, Prony Analysis (PA), Principal Component Analysis, etc^17,18^. Since PMU measurements are usually corrupted with high measurement noise, these methods fail to analyze LFO accurately. EMD technique filters noisy PMU data, but mode mixing is a serious drawback that generates irrelevant modes. Therefore, it becomes very challenging to identify the IMF that represents actual PMU data uncovered from noisy measurements^19^. Ref^20^. utilizes wavelet transform to recover both time and frequency features of an oscillation signal that is very efficient in magnifying the onset of any event. Ref^21^. presents a preprocessing method based on the Teager Kaiser Energy Operator used to check the presence of LFOs, which then utilizes WT with the Yoshida-Bertecco algorithm to identify power system oscillation parameters. However, these algorithms only apply to systems with a single dominant mode^22^. Ref^23^. utilizes the D-space technique to ensure that the critical oscillation modes are placed in a stable region of the complex plane. This approach has been used in several stability analyses and control designs, indicating its effectiveness in managing oscillatory behavior in power systems. Recently, eigensystem realization algorithms (ERA) based on measurement-based power system models have become very popular, where only state matrix A needs to be determined to identify the characteristics of LFO.

Several controllers have also been reported in the literature to suppress these LFOs, including Power System Stabilizer (PSS)^24^, Flexible Alternating-Current Transmission Systems (FACTS) devices^25^, high-voltage direct current (HVDC) links^26^ and wind generation^27^. These approaches utilize a dynamic power system model developed using generator, load, and transmission line datasets. It may not provide enough damping due to inadequate real-time information on the oscillation modes of the system. Consequently, WAMS-based damping of LFO is gaining popularity due to its ability to monitor, locate, and mitigate the effects of LFOs in the power system^15^. Ref^28^. presents a remote feedback controller (RFC) utilizing PMU measurements to dampen inter-area oscillations. Ref^29^. proposes a novel superior PSS that aids local PSS in improving the damping performance of inter-area modes. Ref^30^. proposes a proportional resonant-based PSS to suppress forced oscillation. A detailed discussion regarding the WAMS-based power system damping controller is presented in^31^. In^32^, a decentralized model for power system oscillation damping using PMU signals as feedback signals is presented. In^33,34^, WADC providing supplementary damping control to synchronous generators is discussed. In^35^, a WADC based on WAMS using the Model Linear Quadratic Gaussian (MLQC) method is presented. Ref^36^. utilizes the concepts of mode shape and participation factors, which are critical for developing more effective solutions for enhancing the damping of specific oscillation modes. This paper challenges the idea that installing power system stabilizers is not always the best solution for damping low-frequency oscillations. Ref^37^. emphasizes the critical role of HVDC systems in managing low-frequency oscillations using power modulation strategies. This research contributes to understanding dynamic stability in modern power systems, particularly in the context of increasing interconnectivity and complexity. Ref^38^. provide a comparative analysis with a BESS-based wide-area power system stabilizer, demonstrating that the proposed WADC is more effective in mitigating inter-area oscillations. This comparative approach is common in the literature, as it helps to benchmark new methods against established solutions, providing insights into their relative effectiveness. Ref^15^. introduces a wide-area damping control scheme that utilizes PMU data to dampen selected oscillation modes. This approach is significant as it can achieve effective damping with a minimal number of synchronous generators, which is beneficial for optimizing resources in power systems. Ref^39^. proposes an innovative POD design that simultaneously dampens forced and inter-area oscillations through adaptive and event-triggered control strategies validated by robust testing in a realistic power system environment. Damping both FO and inter-area modes significantly advances over existing methods that focus on one oscillation type. Ref^40^. address the LFO challenges posed by integrating renewable energy sources, particularly large-scale solar photovoltaic (SPV) systems, into existing power grids. WADC provided adequate damping to critical LFO modes, ensuring the stability of power systems under varying operational conditions. The paper employs a Time delay-based feedback controller (TDFC) based approach for the design of the WADC. This method is particularly relevant as it addresses the complexities introduced by time delays and noise in feedback signals, which can severely impact the performance of damping controllers. Ref^41^. introduces a mixed H2/H ∞ control design approach, a sophisticated method used in control theory to ensure robust performance in the presence of uncertainties. This method is particularly relevant in the context of renewable energy sources, as it allows for the design of damping controllers that can handle various operational challenges, including communication delays and system perturbations.

As the literature reveals, inherent time delays in the data transmission from PMUs are an essential factor to consider in the design of WADC. These time delays may affect and destabilize the controller’s performance. However, in the present work, the authors have considered the ideal condition without considering the time delay parameters to solely analyze the controller’s performance.

In this work, authors have presented a methodology to identify and mitigate low-frequency oscillations (Forced and inter-area) using a wide area monitoring system (WAMS) based power system model utilizing phasor measurement units (PMUs). The location of WADC for FO is selected based on generators contributing it using the Improved Ensemble Empirical Mode Decomposition Algorithm with Adaptive Noise (ICEEMDAN), While Eigen system realization algorithm (ERA) is utilized to identify the location of WADC for generators, contributing to inter-area oscillation modes. These approaches provide deep insight into the power system’s behavior in real-time and overcome the method’s dependence on the accurate network model, topology, and parameter values, which are subject to frequent changes. Hence, the source of LFO can be determined accurately to select the locations of PSS to dampen out these oscillations effectively. The performance of CPSS is also improved by introducing a resonant controller, whose resonant frequency is tuned to frequencies of LFOs determined from the Wide Area Power System Model. The damping structure based on PR-PSS with measurements from WAMS effectively suppresses both forced and inter-area oscillation modes. For suppression of FO, the objective is to achieve a significant percentage reduction in its magnitude, while for inter-area oscillation, the objective is to introduce sufficient damping to each dominant mode.

The paper is divided into sections: Section 2 describes the structure of Wide Area Damping Controller, Sect. 3 explains the methods utilized to locate the source of forced and inter-area oscillations. Section 4 describes the working principle of PR-based WADC in mitigating both forced and inter-area oscillation modes. The results and discussions are presented in Sect. 5, while the conclusion is drawn in Sect. 6.

### Wide area damping controller structure

Power systems employ wide-area Damping Controllers (WADCs) to stabilize and dampen low-frequency oscillations. Mitigating these oscillations usually requires coordinating power system device control across a large area. These oscillations can degrade system stability and operational reliability. The WADC utilizes PMUs for real-time wide-area monitoring, offering insights and signals for coordinated control to suppress these oscillations^42^.

**Fig. 1:**
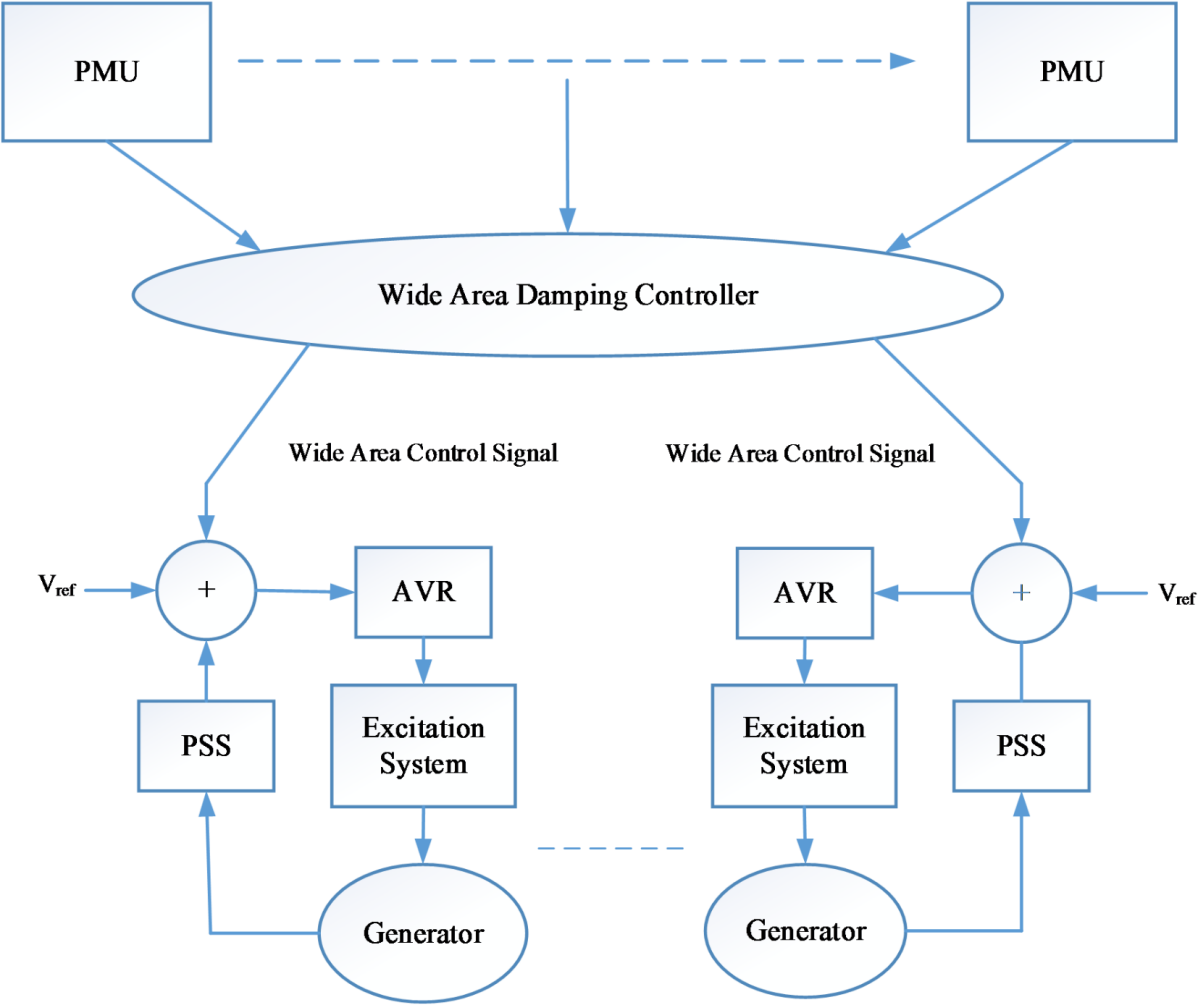
WAMS based WADC Structure.

The simplified structure of WADC shown in Fig. 1 includes the following:

#### Measurement and data acquisition

Wide Area Damping Controllers use real-time data PMUs strategically distributed across the electrical grid. These devices accurately measure voltage, current, and frequency with high accuracy and sampling rates (30–60 Hz), surpassing traditional SCADA systems. They enable improved state estimation and dynamic monitoring, which is crucial for maintaining system stability^43^.

#### Data communication

Real-time PMU data from several locations to a control center requires communication networks. High-speed, reliable communication systems, sometimes employing dedicated infrastructure or the SCADA network, synchronize measurements from different sites. Redundant and secure communication channels prevent data loss and cyber threats^44^.

#### Centralized control center

Phasor Data Concentrators (PDCs) aggregate data from PMUs to enhance grid monitoring and control. They facilitate real-time data processing, ensuring efficient communication and analysis of synchronized data streams. Data is analyzed using advanced control algorithms and signal processing to identify LFO oscillations^44^. The centralized Control Centre performs the following functions:

A. Modeling and Identification: The control center predicts grid behavior using power system models. Real-time data and identification are used to update these models to monitor power system dynamics accurately.
B. Low-frequency oscillation detection and estimation: The control center examines measurements. This involves determining the type of oscillation, frequency, amplitude, and, most importantly, generators contributing to the LFOs. Popular oscillation detection methods include model-based signal processing and spectral analysis^45^.
C. Control Signal Generation: The WADC requires carefully chosen control signals (e.g., generator speed deviations, power flow, or voltage angles) strongly correlated with the targeted oscillatory modes. After detecting power system oscillations, the WADC generates control signals to attenuate them. Power system stabilizers (PSS), flexible AC transmission systems (FACTS), and controllable distributed energy resources (DERs) may receive these control signals^46^.
D. Feedback and Adaptation: The WADC continuously analyzes the power system’s response to control actions. If needed, control parameters are modified in real-time for appropriate damping. This feedback loop keeps the controller stable and adjusts to system changes^46^.

### Low-frequency oscillation detection algorithms

A. FO Detection Algorithm using ICEEMDAN Algorithm.

**Fig. 2:**
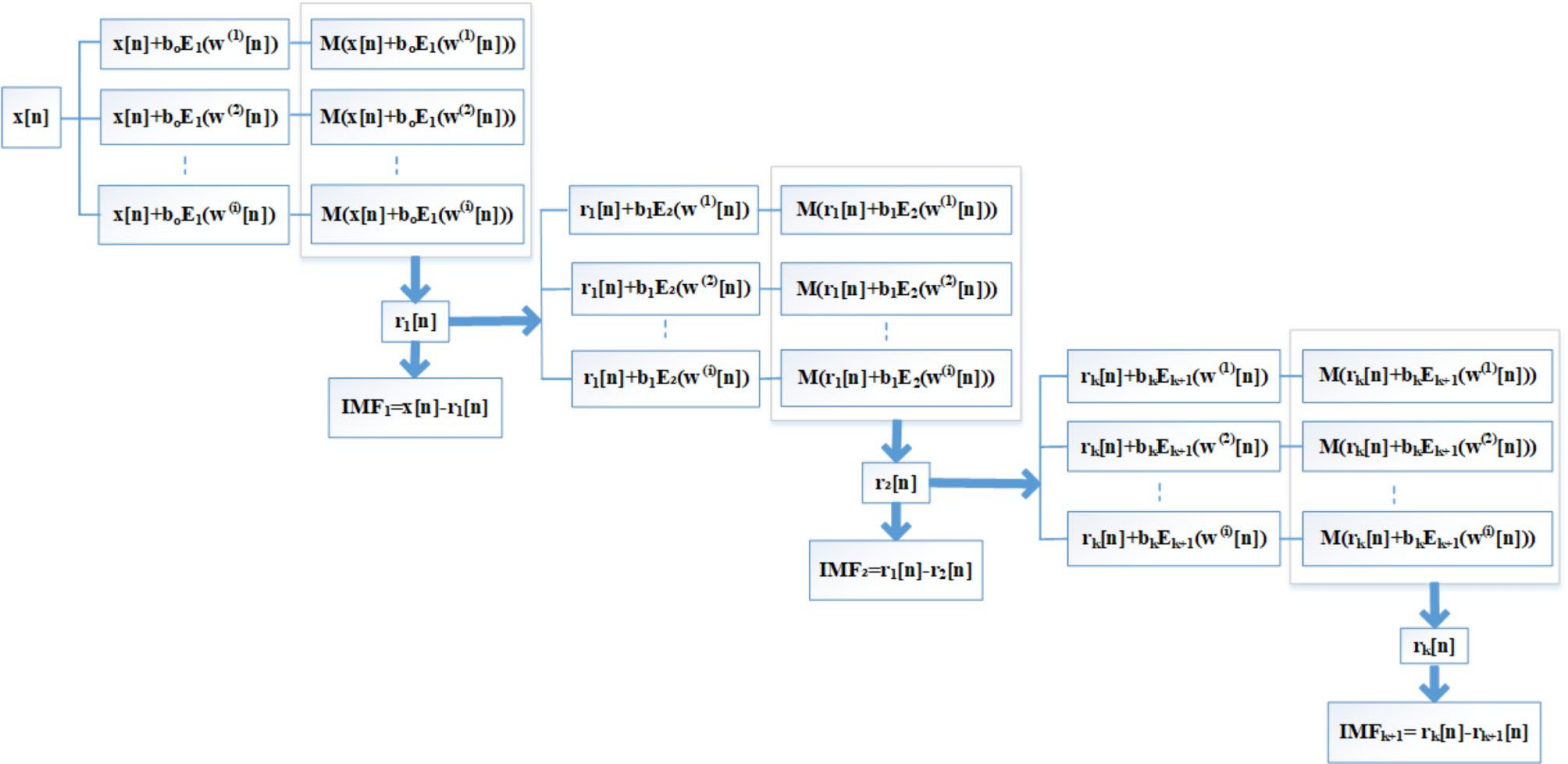
ICEEMDAN Algorithm^45^.

The following steps should be adopted to locate FO:

- Decompose the raw PMU data using the ICEEMDAN algorithm(Fig. 2) to obtain their intrinsic mode functions (IMFs).
- Select the most suitable DMAP that contains the actual signal information based on the correlation coefficient between DMAPs and raw PMU data.
- The oscillation frequency is estimated by segmenting PSDs using the Welch transform. Expressing these segmented PSDs into colormaps gives their time duration.
- Determine the CPSD between the selected DMAP and the raw PMU data from buses associated with generation sources to determine the source location. If a single source causes the oscillations, the amplitude of such a CPSD for that source would be the highest.

B. Inter-Area Oscillation Detection using ERA Algorithm.

**Fig. 3:**
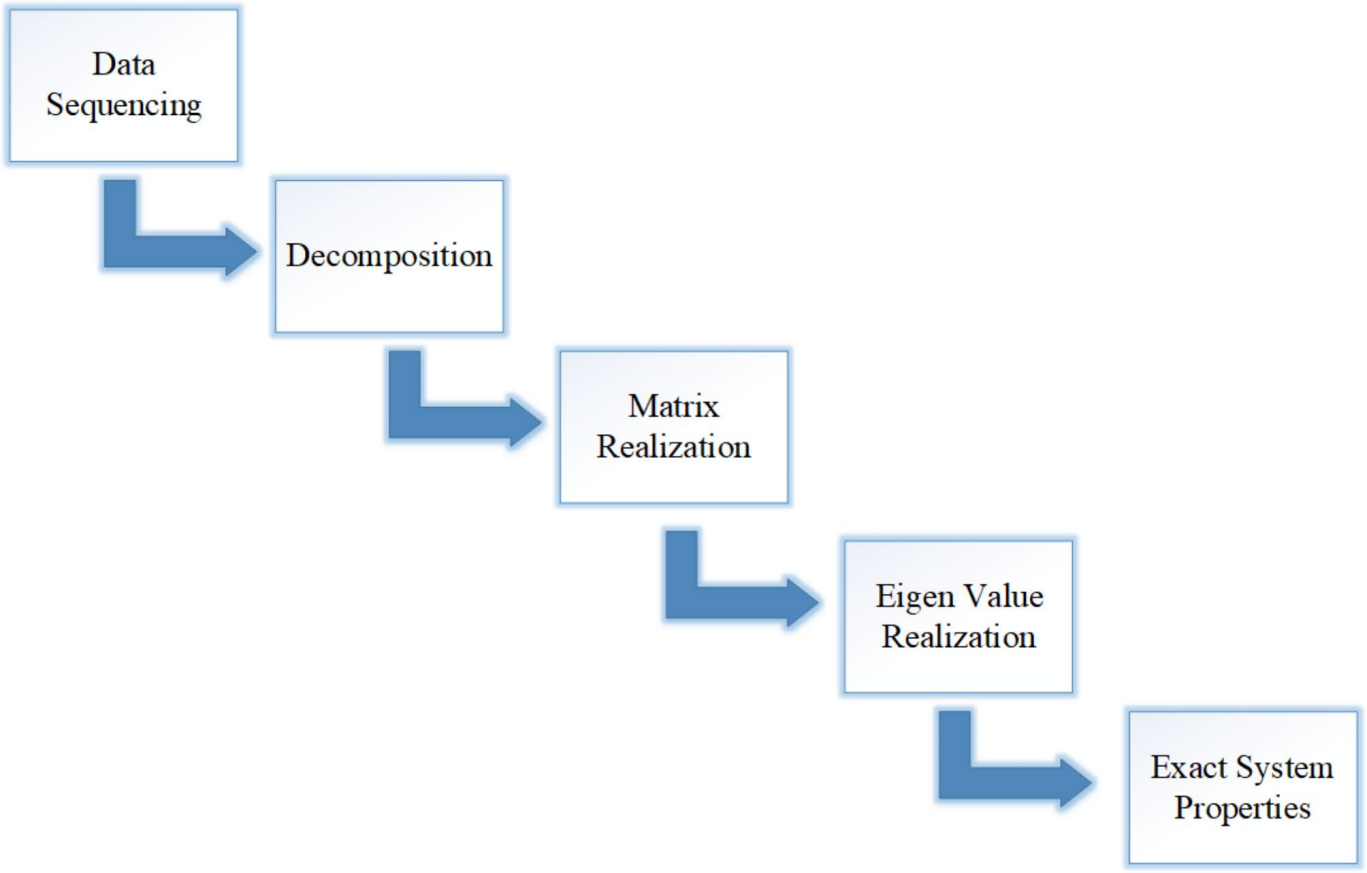
Flow chart of the ERA Algorithm.

The following steps should be adopted to locate the source contributing to inter-area oscillations (Fig. 3) are as follows:

- Given PMU data sequence$$\:\:{a}_{1}$$,$$\:\:{a}_{2}$$$$\:{a}_{n}$$, from multiple locations.
- Compute the hankel matrix H(1) and its time-shifted version H(2) as.

$$H(1) = \:\left[ {\begin{array}{*{20}c} {a_{1} } & {a_{2} } & { \ldots \:} & {a_{n} } \\ {\:a_{2} } & {a_{3} } & { \ldots \:} & {a_{{n + 1}} } \\ {\: \vdots } & \vdots & { \vdots \:} & \vdots \\ {\:a_{n} } & {a_{{n + 1}} } & {\: \ldots \:} & {a_{{n + k}} } \\ \end{array} } \right]$$

$$H(2) = \:\left[ {\begin{array}{*{20}c} {a_{2} } & {a_{3} } & {\: \ldots \:} & {a_{{n + 1}} } \\ {\:a_{3} } & {a_{4} } & { \ldots \:} & {a_{{n + 2}} } \\ {\: \vdots } & {\: \vdots } & {\: \vdots } & \vdots \\ {\:a_{{n + 1}} } & {a_{{n + 2}} } & { \ldots \:} & {a_{{n + k + 1}} } \\ \end{array} } \right]$$

- Decompose the Hankel Matrix using Singular Value Decomposition as.

H (1) = US$$\:{V}^{T}$$

- Extract the new controllability and observability matrix; Calculate the system realization matrix A as.

$$\:\text{A}={S}^{-\frac{1}{2}}{U}^{T}H\left(2\right)\text{V}{S}^{-\frac{1}{2}}$$

- Using the system realization matrix A, compute dominant frequency modes, their corresponding mode shape, and participation factor to identify the generators contributing to low-frequency oscillation.

### Design of PR-based WADC

The primary function of a CPSS is to add positive damping to the generator’s rotor oscillations. This can be done by controlling its excitation using control signals derived from rotor speed, frequency, and electrical power^25^. In the WADC structure, these signals are measured by PMUs. The state-of-the-art structure of CPSS consists of a constant stabilizing gain, washout filter block, lag-lead compensation block, and a limiter. The stabilizing gain Kstab controls the extent of damping provided by CPSS^24^. The washout block is a high-pass filter that bypasses the frequencies related to LFO. The value of its time constant T_w_ ranges between 1 and 20 s. The phase compensation block provides sufficient lead characteristics to counter any phase lag between the exciter input and the generator’s electrical output. The limiter must be used to limit the output of the CPSS to counter the action of the AVR. The values of T_1_ to T_4_ are chosen to provide sufficient phase compensation to achieve the desired damping^47^. The transfer function of CPSS is given by:

1 $$\:\text{G}\text{P}\text{S}\text{S}=\text{K}\text{S}\text{T}\text{A}\text{B}\frac{{T}_{w}s}{1+{T}_{w}s}\:\frac{{1+T}_{1}s}{1+{T}_{2}s}\:\frac{{1+T}_{3}s}{1+{T}_{4}s}$$

It is most widely used in power systems to mitigate inter-area oscillations but cannot effectively reduce forced oscillations. Suppressing FO means minimizing its amplitude. With CPSS, this can be achieved by increasing its gain. However, this method is not practically feasible due to the critical stability gain of the system, which drives the system to unstable states.

Introducing a proportional resonant controller that effectively suppresses both forced and inter-area oscillation modes improves CPSS’s performance.

The transfer function of a PR controller can be written as

2 $${G_{PR}}\left( s \right)\,=\,{K_p}+\frac{{2{K_r}{\omega _c}s}}{{{s^2}+2{\omega _c}s+\omega _{0}^{2}}}$$

Where K_p_ and K_r_ represent the gain of proportional and resonant controllers, respectively. ωc and ωo are the bandwidth and central frequency of the resonant controller.

At the center frequency, we have.

3 $$G_{{PR}} \left( s \right) = K_{p} + {\text{ }}K_{r}$$

Equations 2 and 3 explain the idea of suppressing LFO. At a frequency other than the center frequency, the gain of PR-PSS is equal to Kp and hence acts as a CPSS, while at a center frequency, a high gain equal to K_p_+ K_r_ can be achieved without affecting the performance of CPSS.

**Fig. 4:**
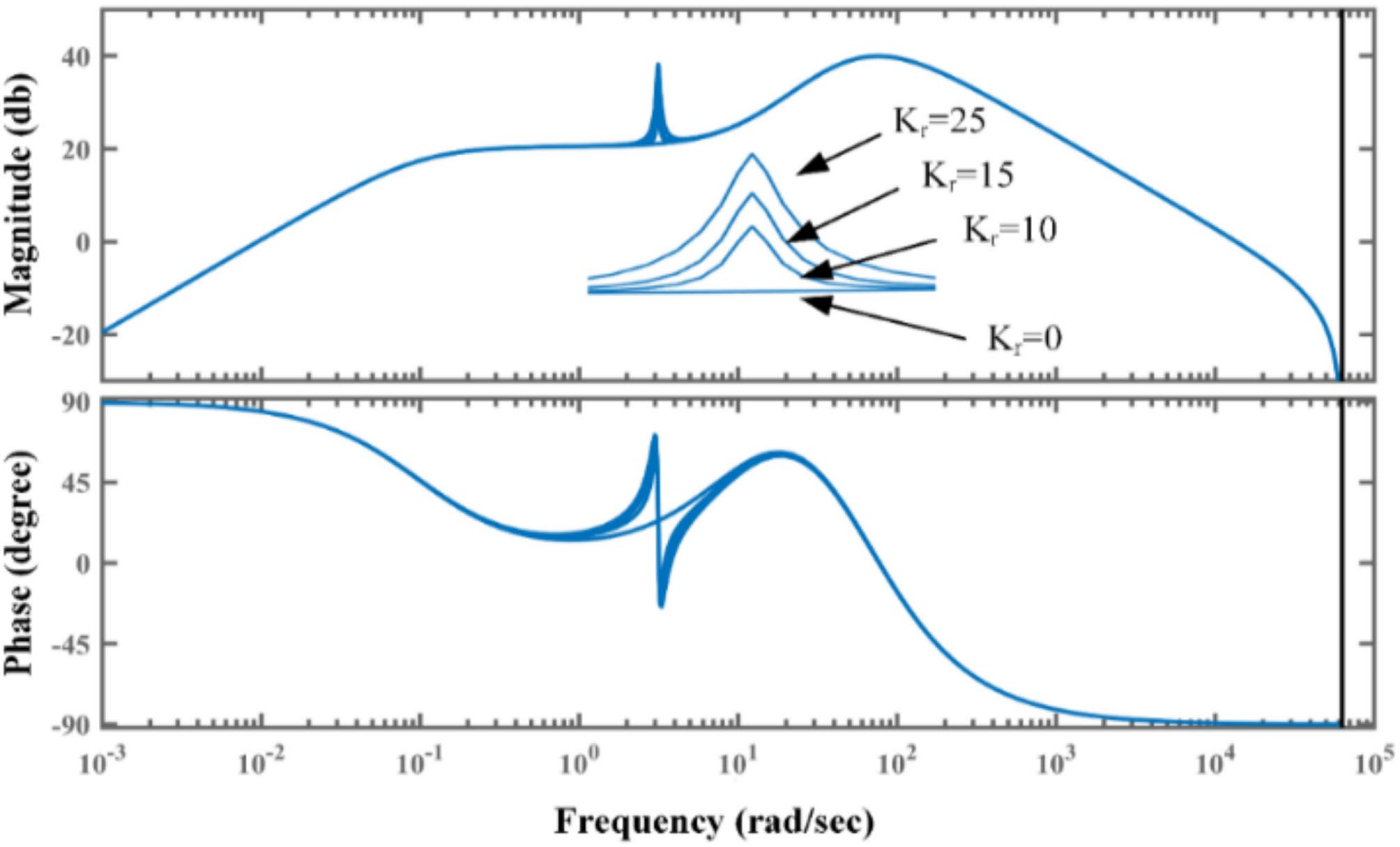
Bode plot of PR-PSS with different values of proportional gain Kr.

**Fig. 5:**
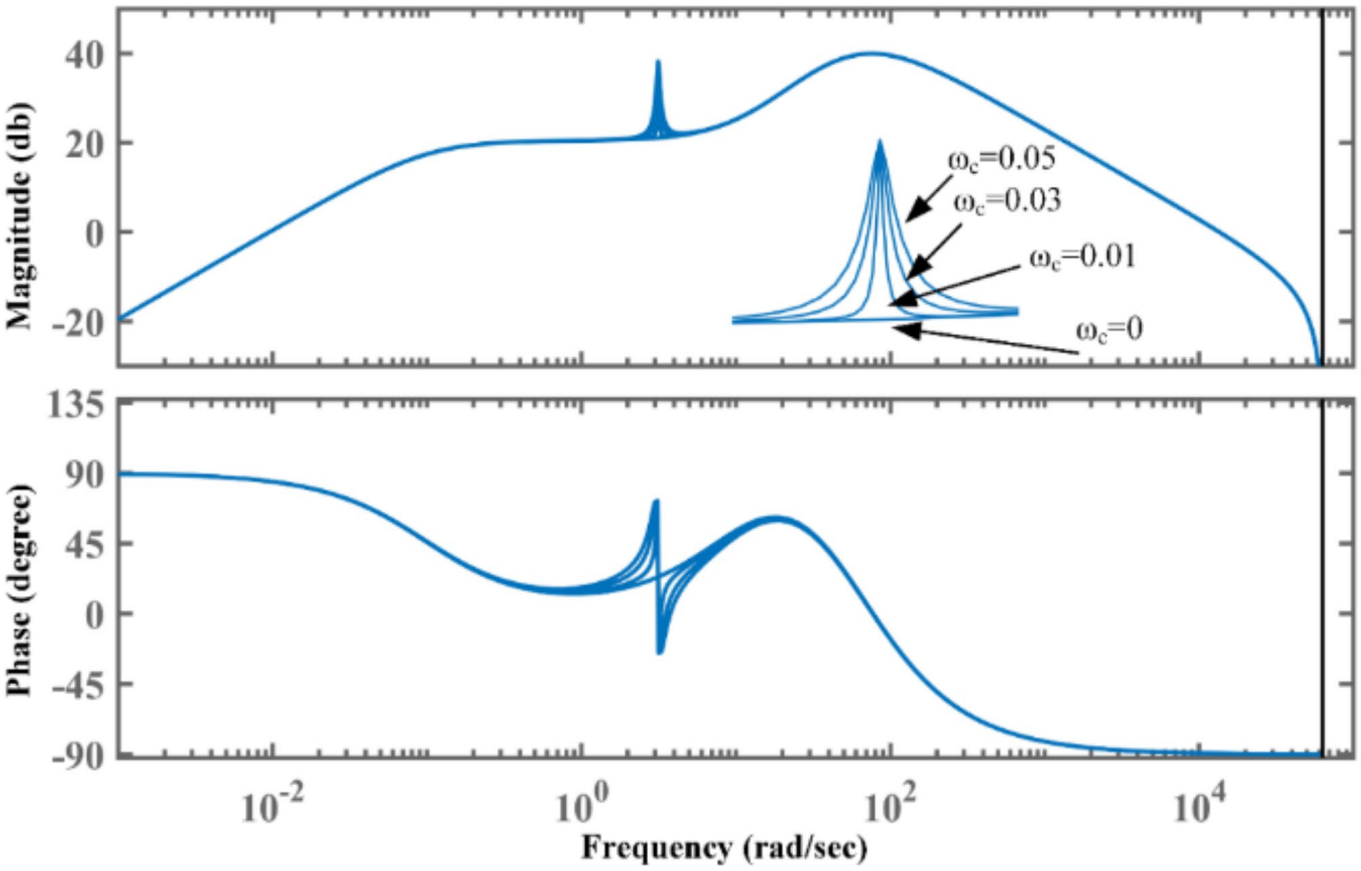
Bode plot of PR-PSS with different values of ω_c_.

This can be easily understood using a bode plot of PR-PSS, as shown in Fig. 4. By selecting the high value of Kr, significant gain at a central frequency can be obtained without affecting the performance of the CPSS. With Kr = 0, the PR-PSS behaves similarly to CPSS. In addition, phase compensation remains unaltered with introducing a PR controller. Therefore, its introduction improves the performance of CPSS in mitigating LFO. Although bandwidth ω_c_ does not affect the gain of PR-PSS, its higher values enable a wider operating range to tackle variations in frequencies of LFO. Figure 5 shows the bode plot of PR-PSS with an increasing value of ω_c_ with constant Kr. It can be observed from the bode plot both magnitude and phase response are similar to CPSS with ωc = 0. At the same time, its range around center frequency becomes broader for higher values of ω_c_ to cope with slight variations in frequencies of LFO for its efficient mitigation.

**Fig. 6:**
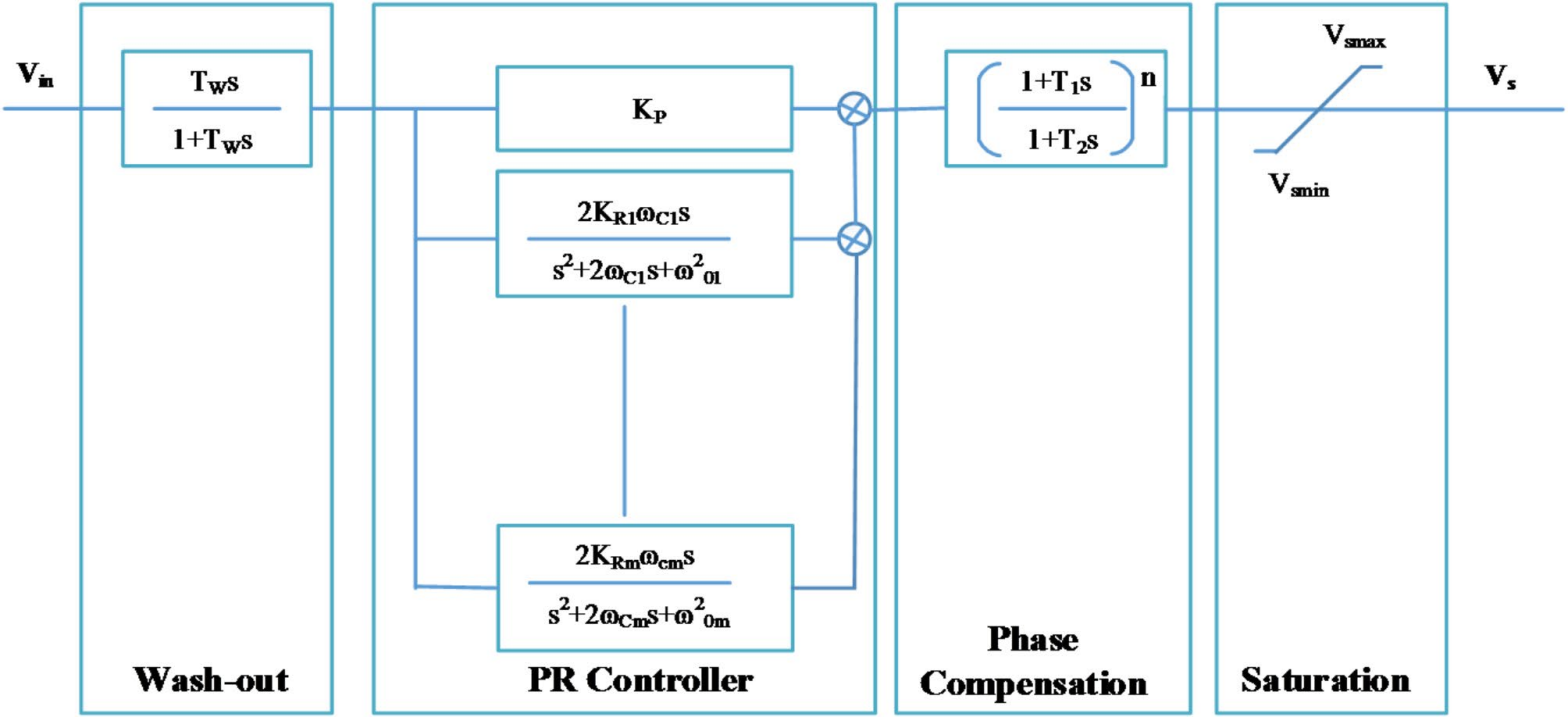
Proportional Resonant Power System Stabilizer.

Since several sources of FO or inter-area modes might be present in the system, multiple bands of resonant controllers are used in parallel to tune their frequency to the frequencies of LFO modes present in the system. The block diagram of Multi-band PR-PSS is shown in Fig. 6. Its transfer function can be written as

4 $$\:\text{G}\text{P}\text{R}-\text{P}\text{S}\text{S}=Kstab\frac{{T}_{w}s}{1+{T}_{w}s}\:\text{G}\text{P}\text{R}\left(\text{s}\right)\:\frac{{1+T}_{1}s}{1+{T}_{2}s}\:\frac{{1+T}_{3}s}{1+{T}_{4}s}$$

Where G_PR_ is the gain of a multi-band PR controller. Its transfer function can be written as.

5 $$GPR(s) = KP + \sum\nolimits_{{(i = 1)}}^{m} {\frac{{2K_{{Ri}} \omega _{{ci}} s}}{{s^{2} + 2\omega _{{ci}} s + \omega _{{0i}}^{2} }}}$$

The parameters for the PR-PSS are determined based on the oscillation frequencies present in the system. The design involves Setting the resonant controller parameters (K_R_, ω_c_, ω_0_) to match the frequencies of the oscillation modes that need suppression. The method includes analyzing the system’s frequency response to ensure that the PR-PSS can effectively dampen the oscillations without adversely affecting system stability. The design must ensure that the stability of the power system is not compromised while effectively damping the oscillations. This is crucial as increasing the gain too much can lead to instability.

## Result and discussion

**Fig. 7:**
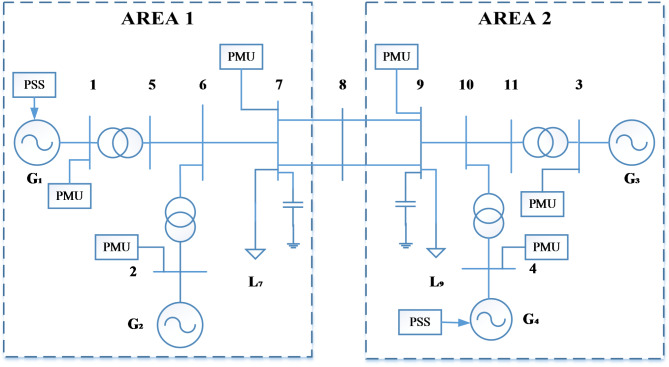
Two-Area Four-Machine System.

In this study, the performance of the WAMS-based PR-PSS is analyzed on an area four-machine system. The simulation is carried out using the Matlab 2018 version. The inbuilt PMU block was inspired by the IEEE Std C37.118.1–2011 utilizing a Phase-Locked Loop (PLL) algorithm to estimate voltage magnitude, phase angle (relative to PLL phase), and frequency. However, the model does not explicitly mention latency considerations. The PMUs are installed on all the generation buses, including buses 7 and 9, as shown in Fig. 7. The FO cases are generated by modulating the shaft torque using a sinusoidal signal with a peak amplitude of 0.1. Inter-area oscillations are generated by random load fluctuations or by creating three-phase faults. The performance of PR-PSS is analyzed in terms of percentage reduction in magnitude for FO cases while for inter-area oscillations in terms of the percentage improvement in damping ratio.

**Fig. 8:**
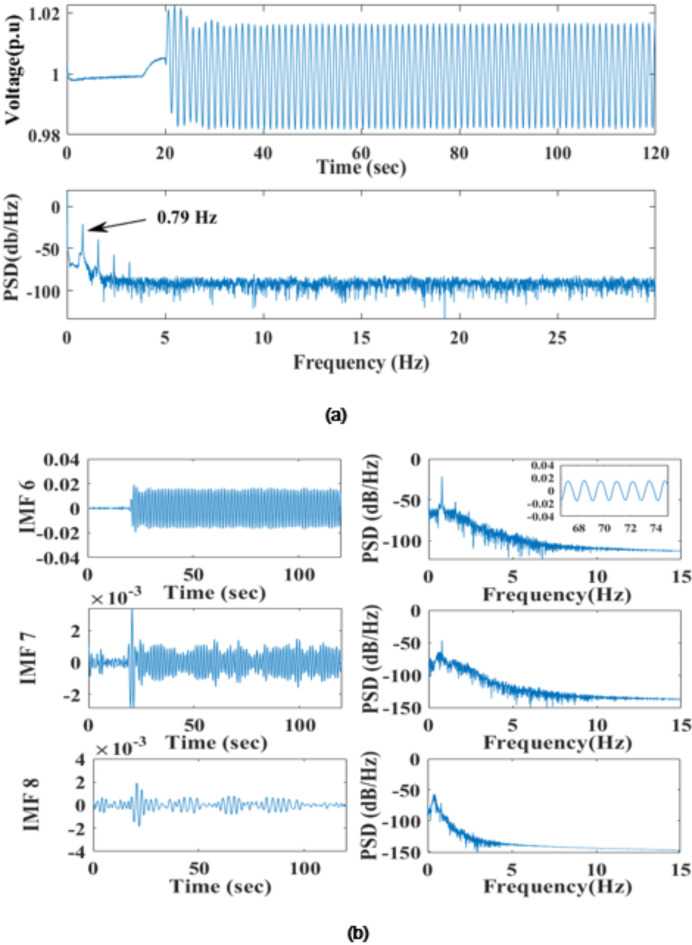
(**a**) Noisy PMU data at Bus 7 (**b**) Decomposed signal with their PSD having the highest Correlation coefficient with actual PMU data.

**Fig. 9:**
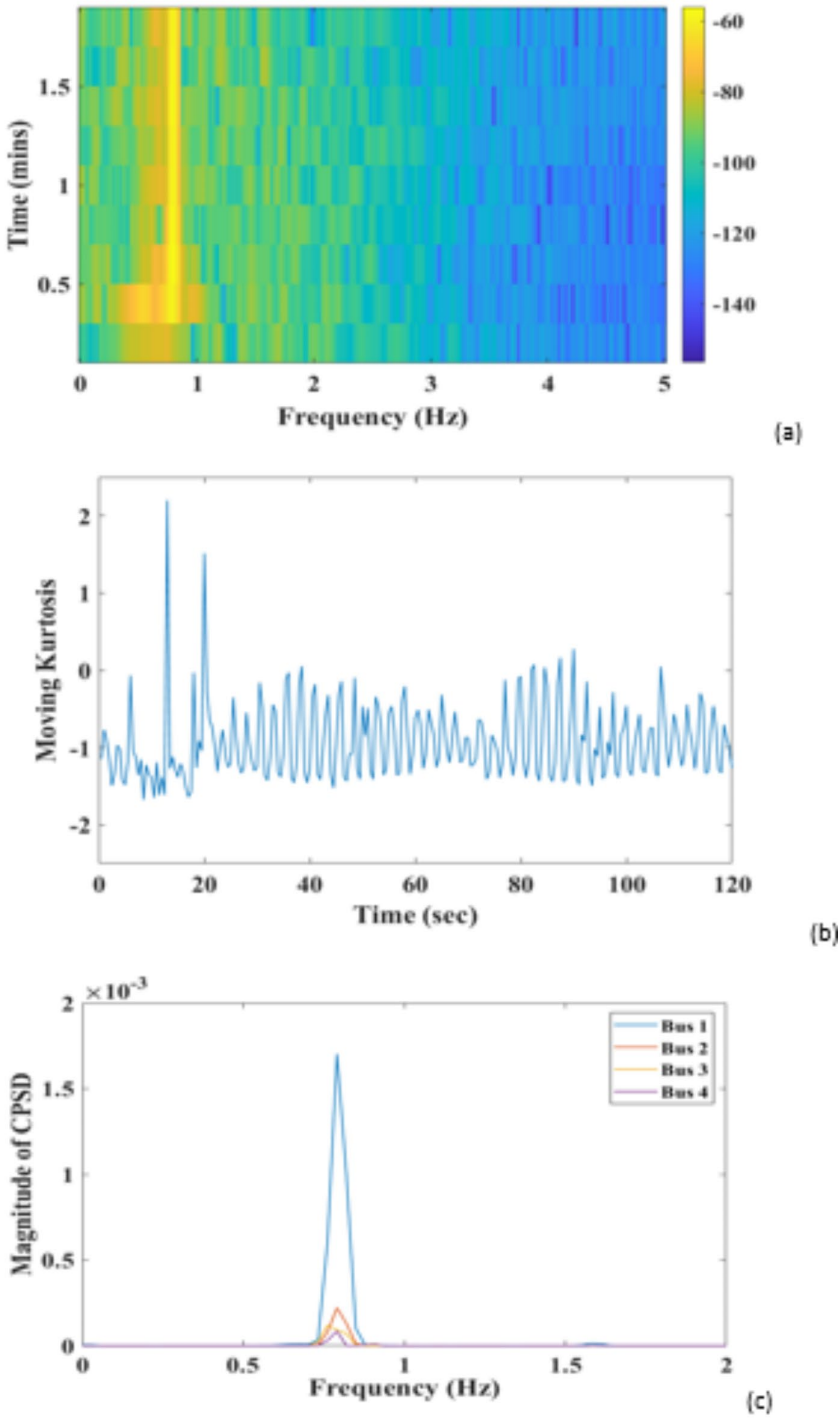
(a) Colormap showing the duration of FO (**b**) Moving Kurtosis determining the type of oscillation (c) CPSD of chosen DMAP and raw PMU data from generation sources.

For FO oscillation, noisy PMU data having multiple frequency components (bus 7) is decomposed using ICEEMDAN Algorithm (Fig. 8(a)). The signal is decomposed into 12 modes. IMF 6 has the highest correlation coefficient of 0.78, making it the most relevant DMAP for analysis (Fig. 8b). The segmented PSD identified FO with a unique peak of 0.79 Hz, which suggests the effectiveness of the proposed method. Figure 9a shows a segmented PSD colormap that confirms the sustained oscillation lasts 20–120 s. Figure 9(b) shows sustained oscillation is a FO between 20 and 120 s; for identification of the oscillation source, CPSD is calculated between selected DMAP and bus associated with the generation source. Figure 9(c) shows that bus 1 has the highest CPSD at 0.79 Hz, associated with G1. Therefore, the identified location of FO is at generator G1; PR-PSS is placed at G1 to dampen the effect of these oscillations.

**Fig. 10:**
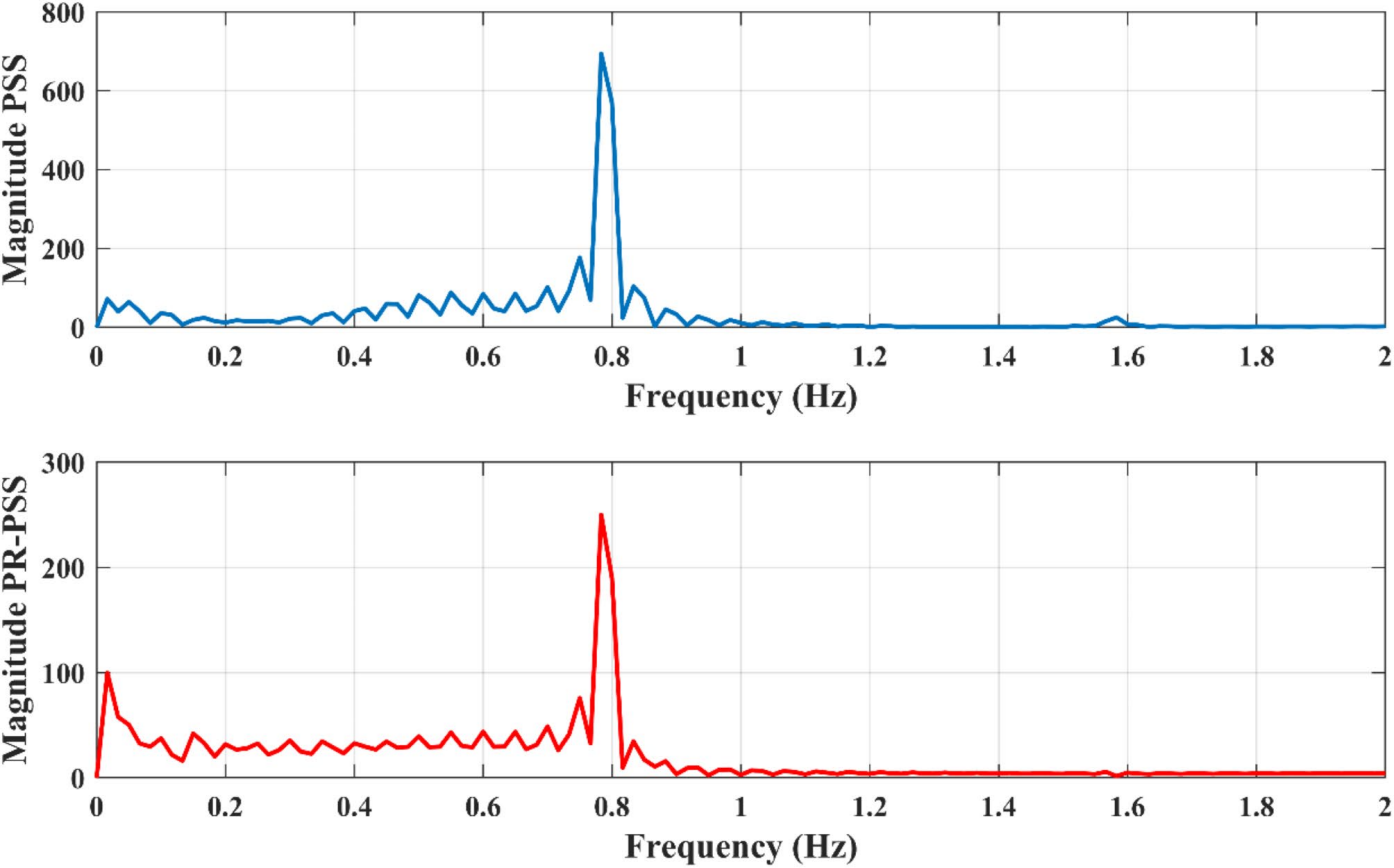
FFT for FO signal with different values of Kr.

**Fig. 11:**
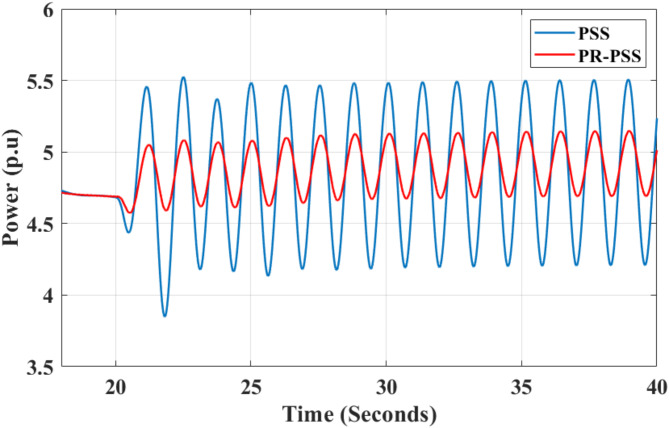
Time response of active power between bus 7 and 9.

**Table 1:** Percentage amplitude reduction in FO with different values of K_r_.

S.No	Frequency (Hz)	K_*r*_	Peak value from FFT response	% Reduction
1	0.7925	0	693.8	0
2	0.7856	25	526.15	24.16
3	0.7910	50	424.65	36.84
4	0.7926	100	249.99	63.96

The parameters of PR-PSS are K_p_=K_s_, K_r_=100, ω_c_ = 0.05 and wo = 2π$\:\times\:$0.79 with all other parameters remain same as in CPSS. The tie-line power between bus 7 and bus 9 is observed and compared with CPSS. To estimate the percent reduction in the magnitude of FO with PR-PSS compared to CPSS, the FFT of both signals are constructed, and their maximum values are compared to determine the amplitude scaling provided by PR-PSS. For Kr = 0, the maximum value obtained from FFT is 693.80, while for Kr = 100, the maximum value of FFT is shown in Fig. 10. The results obtained for different values of K_r_ are given in Table 1. It can be observed that the magnitude of forced oscillations is substantially reduced with the introduction of PR-PSS with a high value of Kr, as shown in Fig. 11. The amplitude response of FFT for FO data gives an idea about the severity of the oscillation and determines if the system’s response exceeds safe operational limits. Understanding the FFT peak allows engineers to design systems that either avoid resonance conditions or intentionally utilize them (e.g., in resonators or oscillatory systems) while managing the amplitude and damping for stability and performance.

**Fig. 12:**
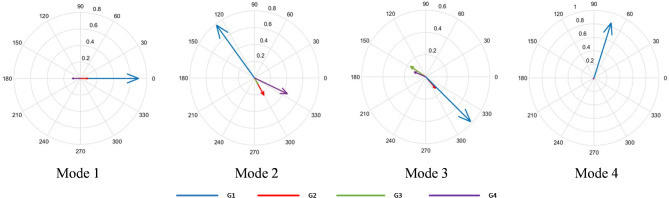
Estimated Mode Shapes.

**Table 2:** Results of modal analysis.

Mode	Participation Factor				Mode Shape
	G_1_	G_2_	G_3_	G_4_	
1	0.72	0.09	0.13	0.32	G_1_ Vs G_4_
2	0.58	0.06	0.09	0.39	G_1_ Vs G_2_,G_3_
3	0.53	0.03	0.51	0.12	G_1_ Vs G_2_,G_3_
4	0.69	0.03	0.04	0.19	G_1_ Vs G_4_

**Table 3:** Effect of PR-PSS on inter-area modes.

Mode	CPSS		PR-PSS	
	Frequency(Hz)	Damping Ratio	Frequency(Hz)	Damping Ratio
1	0.6432	0.0627	0.6475	0.0956
2	0.3724	0.0912	0.3812	0.1126
3	1.0647	0.0496	1.0956	0.0725
4	0.5612	0.0735	0.5614	0.0978

For the identification of inter-area modes, the frequency data at Bus 7 and 9 are selected to develop a measurement-based power system model to estimate system Matrix A. System Matrix A is utilized for model analysis parameter estimation. The stacking factor s ranges from 200 to 450 for all cases, and the data window for the augmented Hankel matrix is 10 s.

Figure 12 plots four inter-area modes with less than 10% damping ratios using the estimated left and right eigenvectors. Mode shape 1 shows that Area 1 generator G1 oscillates against Area 2 generator G4, with G1 playing a significant role. Mode shape 2 shows that Area 1 generators G1 and G2 and Area 2 generators G3 and G4 oscillate coherently. Generators G1 and G4 influence this oscillation. Mode shape 3 shows G1 and G3 oscillate against each other with a high participation factor. Mode 4 indicates that Generator G1 in Area 1 oscillates against Generator G4 in Area 2 with high participation. The results of the participation factor and mode shapes of each generator contributing to the dominant oscillation mode are given in Table 2. Based on the results, the optimal location for placing the PR-PSS controller is at generators G1 and G4.

**Fig. 13:**
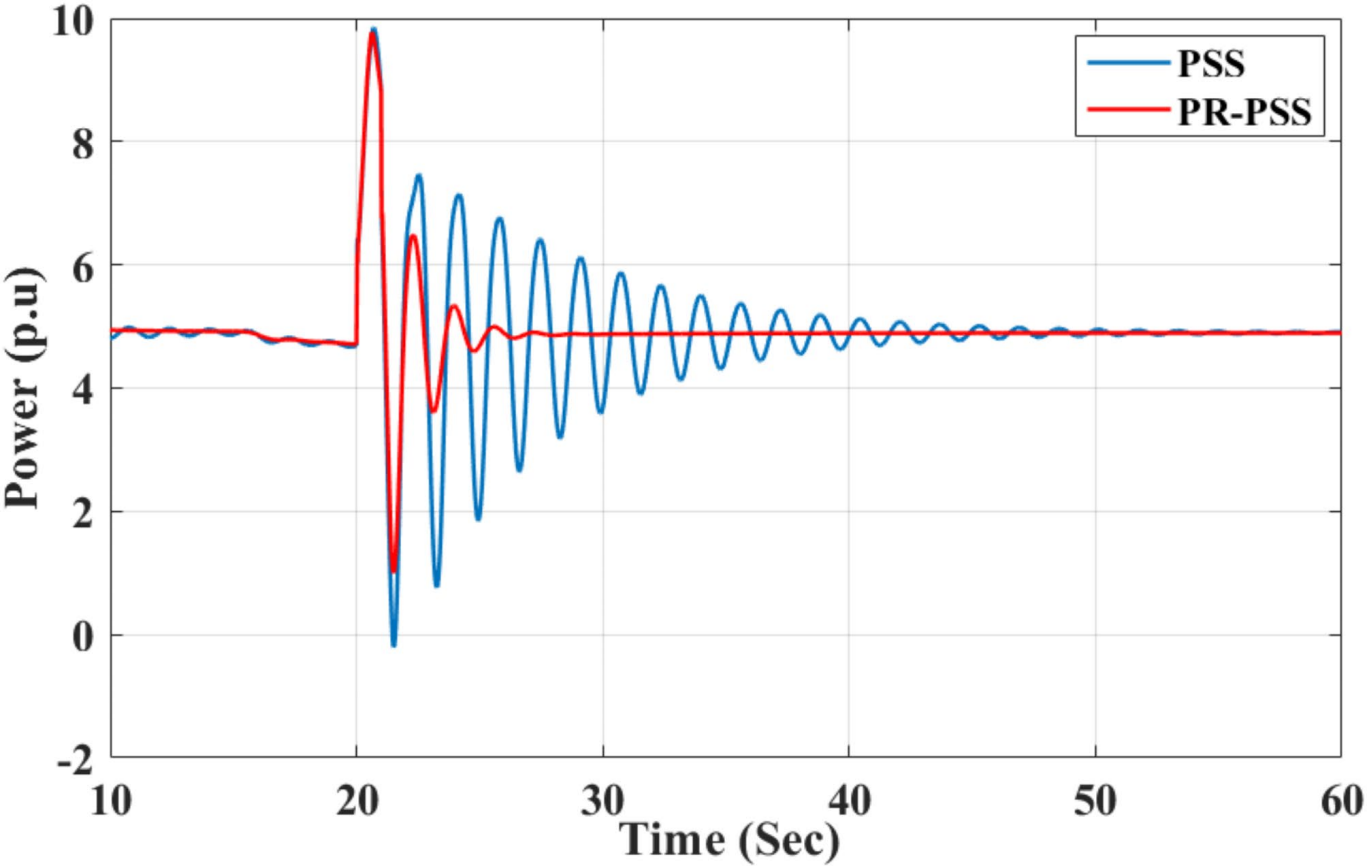
Time response of active power between buses 7 and 9.

For the sake of simplicity, the value of Kr is kept equal to 50 for all the multi-band controllers considered. The values are the frequency of resonant controllers equal to the frequency of dominant oscillation modes tabulated in Table 2. Although the CPSS performs satisfactorily to damp out inter-area modes, adding a PR controller further improves its performance, as shown in Fig. 13. The improvement in the damping ratio of dominant oscillation modes is given in Table 3.

The results show the capability of the PR-PSS to suppress multimode forced and interarea oscillations, which arise from different sources, such as mechanical power fluctuations and load variations. Using multiple resonant controllers in parallel allows the PR-PSS to effectively mitigate these oscillations until it lies within the bandwidth frequency range over which the controller is effective, suggesting that it can be robust across various operational scenarios.

Compared to the results obtained in^39^, both methods effectively mitigate forced and inter-area oscillations. However, both techniques are entirely different. One uses an adaptive power oscillation damper, effective even with communication uncertainties, while the considered method evaluates the performance of a Proportional-Resonant Power System Stabilizer (PR-PSS) in a complex power system, demonstrating its effectiveness in improving damping ratios for both single-mode and multimode forced oscillations caused by mechanical power fluctuations and load variations.

The proposed work can be further extended by considering wind and photovoltaic generation in the system. Wind and photovoltaic generation dynamics differ significantly from those of synchronous generators. Wind turbines and PV systems are often interfaced with the grid through power electronics, which can introduce different oscillation characteristics and stability issues not addressed in the current test system. While the current test system may not directly apply to wind and PV generation, the principles of the proposed PR-PSS could still be relevant. Future research could adapt the methodology to include renewable energy sources by developing models that account for the unique dynamics of these systems. Another research direction includes consideration of inherent time delays in the data transmission from PMUs in the design of WADC. These time delays may affect the controller’s performance and destabilize it.

## Conclusion

Low-frequency oscillations are now very common in modern large interconnected power systems. Timely detection, accurate source identification, and mitigation of these oscillations are essential for reliable system operation. A two-step PMU-based method is used to locate FO sources, while the measurement-based power system model based on the ERA algorithm is utilized to locate the source of inter-area oscillations. The mitigation of LFO can be done by increasing the stabilizing gain of CPSS. However, it can be increased up to a certain level due to the limits imposed by the critical stability gain of the system, after which the system cannot maintain its stability. This problem is overcome by adding a resonant controller to CPSS, which increases the gain at the corresponding oscillation frequency without affecting the critical stability gain of the system. The performance of the proposed method is tested on the two-area, four-machine system under different cases of LFO. The results show the superiority of PR-PSS over CPSS in suppressing both forced and inter-area oscillations. However, choosing an appropriate gain for each band of the resonant controller is challenging to obtain better results.

## Data Availability

The datasets used and/or analysed during the current study available from the first author on reasonable request.

## Abbreviations

PMU: phasor measurement unit
PDC: phasor data concentrator
SCADA: supervisory controller and data acquisition
WAMS: wide area monitoring system
WADC: wide area damping controller
LFO: low-frequency oscillations
FO: forced oscillations
CPSS: conventional power system stabilizer
PR-PSS: proportional resonant-based power system stabilizer
FFT: fast fourier transform
PA: prony analysis
EMD: empirical mode decomposition
IMF: intrinsic mode function
WT: wavelet transform
FACTS: flexible alternating-current transmission systems
MLQC: model linear quadratic gaussian
TDFC: time delay-based feedback controller
ICEEMDAN: improved ensemble empirical mode decomposition algorithm with adaptive noise
ERA: eigensystem realization algorithm
